# Serial casting in early onset scoliosis: syndromic scoliosis is no contraindication

**DOI:** 10.1186/s12891-019-2938-9

**Published:** 2019-11-20

**Authors:** Tobias M. Ballhause, Menard Moritz, Annika Hättich, Ralf Stücker, Kiril Mladenov

**Affiliations:** 1 Department of Pediatric Orthopaedic Surgery, Children’s Hospital Hamburg-Altona, Hamburg, Germany; 2 Department of Trauma, Hand and Reconstructive Surgery, University Medical Center Hamburg-Eppendorf, Martinistr, 52, 20246 Hamburg, Germany; 3 Department of Orthopaedic Surgery, University Medical Center Hamburg-Eppendorf, Hamburg, Germany

**Keywords:** Early onset scoliosis (EOS), Infantile scoliosis, Syndromic-associated EOS, Serial casting

## Abstract

**Background:**

Serial casting is a treatment for early onset scoliosis (EOS) in young children to achieve curve correction before bracing or to postpone initial surgical treatment until the patient is older. Good results have been reported for patients with idiopathic early onset scoliosis (IS). However, there are few reports of results in non-idiopathic cases, and the benefits of non-surgical methods in the syndromic-associated early onset scoliosis subgroup are unknown.

**Methods:**

Retrospective single-institution study of patient charts and X-rays of all cases of sustained serial casting for EOS.

Staged correction was obtained by applying three consecutive casts under general anaesthesia. These were changed every 4 weeks, followed by the implementation of a custom-made full-time Chêneau brace. Correction was measured by Cobb angle (CA) and rib-vertebra angle difference (RVAD) on whole spine anterior-posterior radiographs. Statistical analysis was performed via ANOVA.

**Results:**

The study group consisted of 6 patiens with IS and 10 with non-idiopathic scoliosis (NIS) – exclusively syndromic-associated. The mean age at onset of treatment was 35 months (±15). The mean follow up was 21 months (±15). In IS patients average CA/RVAD before treatment was 46°(±8)/20°(±12). In NIS patients average CA/RVAD before treatment was 55°(±15)/24°(±14). After application of the third cast, the CA/RVAD was reduced to 20°(±11)/11°(±10) in IS patients. Whereas in NIS patients average CA/RVAD after the thrid cast was 28°(±12)/18°(±13). At latest follow-up the CA/RVAD was 16°(±7)/9°(±8) in IS patients and 31°(±11)/17° (±15) in NIS patients.

**Conclusion:**

Syndromic etiology is not a contraindication for serial casting in EOS. Our results show a curve correction, measured in CA, of 65% in IS patients and 44% in NIS patients. Significant reduction in the morphologic deformity, measured in RVAD, was achieved in the IS cohort, but not in the NIS cohort. In all cases surgical treatment could be delayed.

## Background

Early onset scoliosis (EOS) includes all forms of scoliosis that present within the first 10 years of life [1]. In contrast to adolescent scoliosis, EOS is mostly of non-idiopathic origin and can be caused by congenital vertebral anomalies and neuromuscular or syndromic diseases.

The growth rate within the first 12 months of life is the most rapid in a human’s lifetime. Major spinal deformity might lead to a thoracic insufficiency syndrome [2]. A 33% prevalence of obstructive lung disease was observed in children with syndromic or congenital scoliosis [3]. In order to preserve patient’s pulmonary capacity, EOS needs to be treated during early childhood.

Growing rod constructs are currently the treatment of choice for severe curves or for deformities that do not respond to non-operative treatment. However, despite the positive effects that growing rod constructs provide, an initial surgical procedure in children under the age of 4 years increases the risk of complications [4]. Serial corrective casts with consecutive bracing have proven to be a safe and adequate method for the treatment of EOS patients [5, 6]. Moreover, the consecutive Chêneau brace tends to be better accepted by younger children compared to juveniles. A growth-correcting procedure established during early childhood has the most potential for correction. Mehta described growth itself as a “corrective force” [7]. Good results with serial Risser casts have been reported in patients with idiopathic early onset scoliosis (IS) [8]. Only few studies of serial Risser casting in EOS patients with non-idiopathic scoliosis (NIS) have been published. The benefits of nonsurgical methods in patients with syndromic-associated EOS as a subgroup of EOS are poorly defined. Furthermore, treatment protocols for nonsurgical methods vary significantly in the literature, especially in the number of castings and the wear time [9, 10].

The purpose of this study is to describe our treatment protocol and to evaluate the results after serial casting for patients with early onset scoliosis and compare the results in relation to the background of the scoliosis.

## Methods

**Fig. 1:**
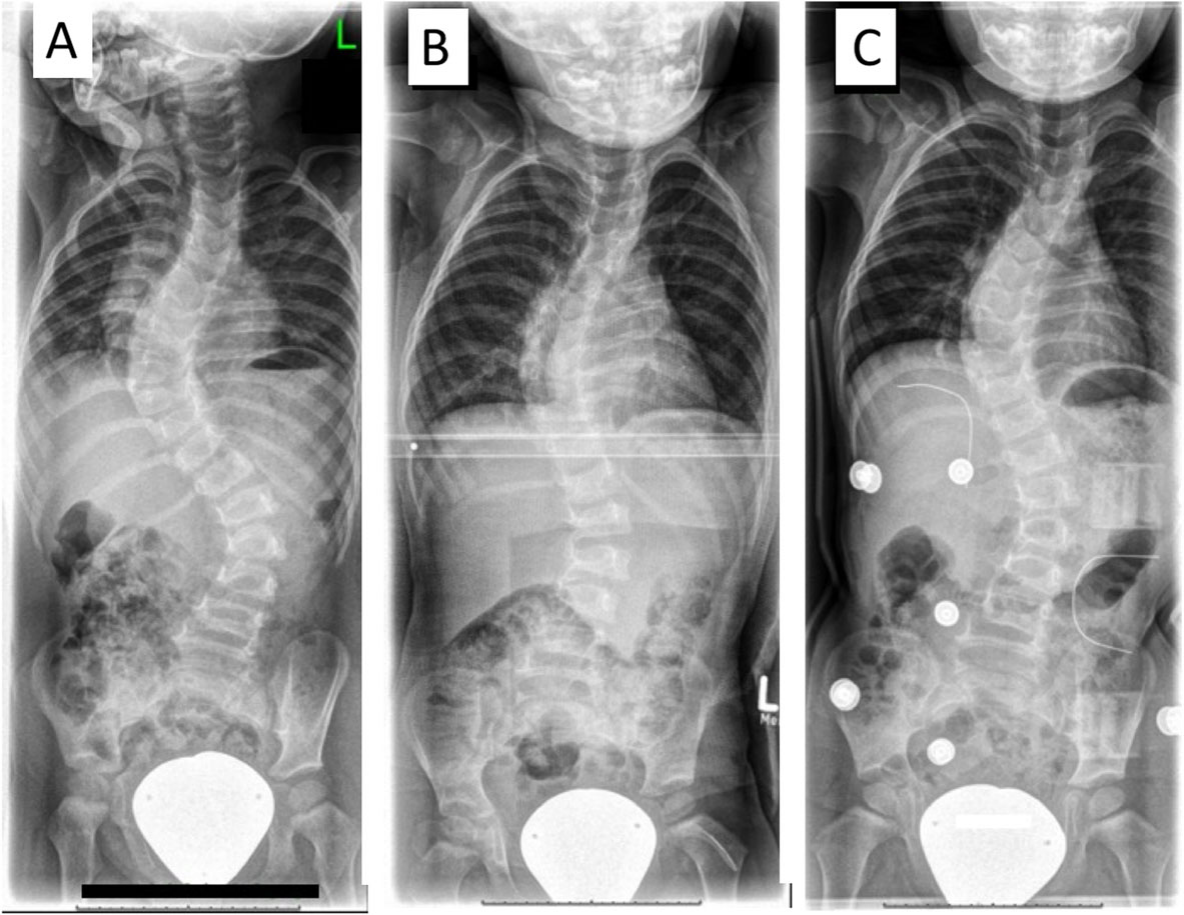
Consecutive radiographs showing the spinal column of the same patient before treatment in the first cast and in brace. **a** The spinal column before casting, CA = 66°. **b** Radiograph after implementation of the first Risser cast. The curve was reduced to 42°. **c** After the third Risser cast the Chêneau brace was applied, the remaining CA = 39° in the brace

Retrospective single-institution study of patients with EOS of idiopathic or syndromic origin. According to etiology, patients were divided into two groups (IS and NIS). Patient data were obtained from charts and radiographs. To be included in the study patients had to be under the age of 6 years, received three serial casts and after casting a Chêneau brace. The minimum follow-up was 6 months. The following radiologic parameters were measured: Cobb angle (CA), rib-vertebra angle difference (RVAD), and phase of rib-vertebra relationship according to Mehta. These parameters were evaluated at the onset of treatment, after each cast application, and at the time of latest follow-up (Fig. 1).

Statistical analysis was performed using GraphPad Prism 8 (*GraphPad Software, La Jolla, CA, USA*). Multiple comparisons were performed via mixed ANOVA. Significance was set at *p* < 0.05.

Syndromes were verified by genetic testing (Table 1). One patient had an unknown syndrome.

**Table 1:** Characteristics of collectivity

Patient	Age range in months	Rib-Phase	Origin of Scoliosis
1	12–24	2	IS
2	12–24	1	IS
3	49–60	1	IS
4	49–60	2	IS
5	25–36	2	IS
6	25–36	2	IS
7	12–24	2	CREBBP mutation
8	37–48	1	Williams-Beuren syndrome
9	49–60	1	monosomy 6q25
10	49–60	2	CASK syndrome
11	37–48	2	Prader-Willi syndrome
12	37–48	1	unspecified syndrome
13	12–24	2	Smith-Lemli-Opitz syndrome
14	12–24	2	Williams-Beuren syndrome
15	12–24	2	Marfan syndrome
16	25–36	1	Prader-Willi syndrome

All patient with the subjection of a syndromic disease consulted a geneticist for specific testing. Only one patient had an unknown syndromic disease

**Fig. 2:**
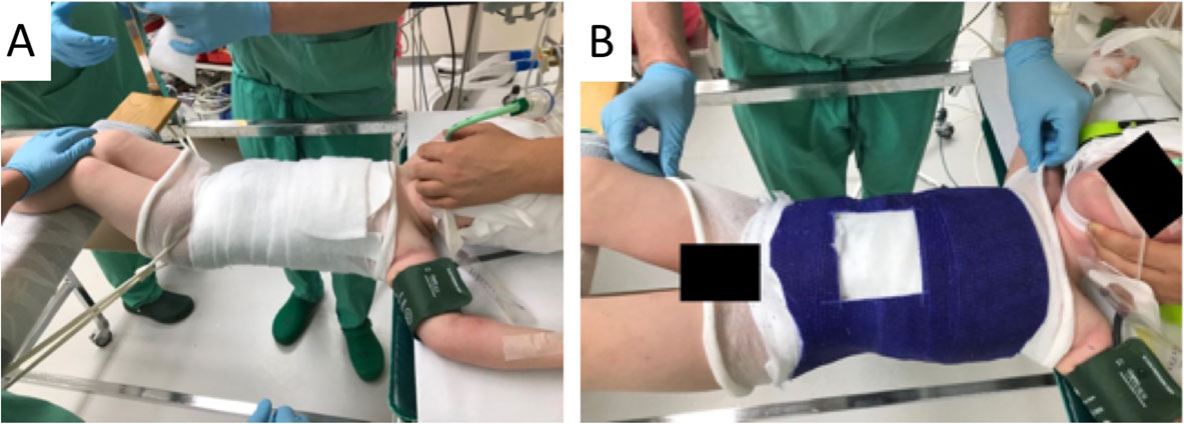
Application of a Risser cast under general anesthesia. The procedure is performed on a Risser’s table. **a** The complete trunk is padded, and the belly region is cushioned. **b** Fiberglass cast tape is wrapped around the trunk. It is modulated under derotation and lateral pressure to the thorax and abdomen. A longitudinal pull is applied during the entire procedure by two assistants holding the arms/shoulders and the legs/feet. In order to facilitate breathing, a window is sawed in the cast’s abdominal region. The cast is individually adjusted by sawing curves for thighs and upper arms

**Fig. 3:**
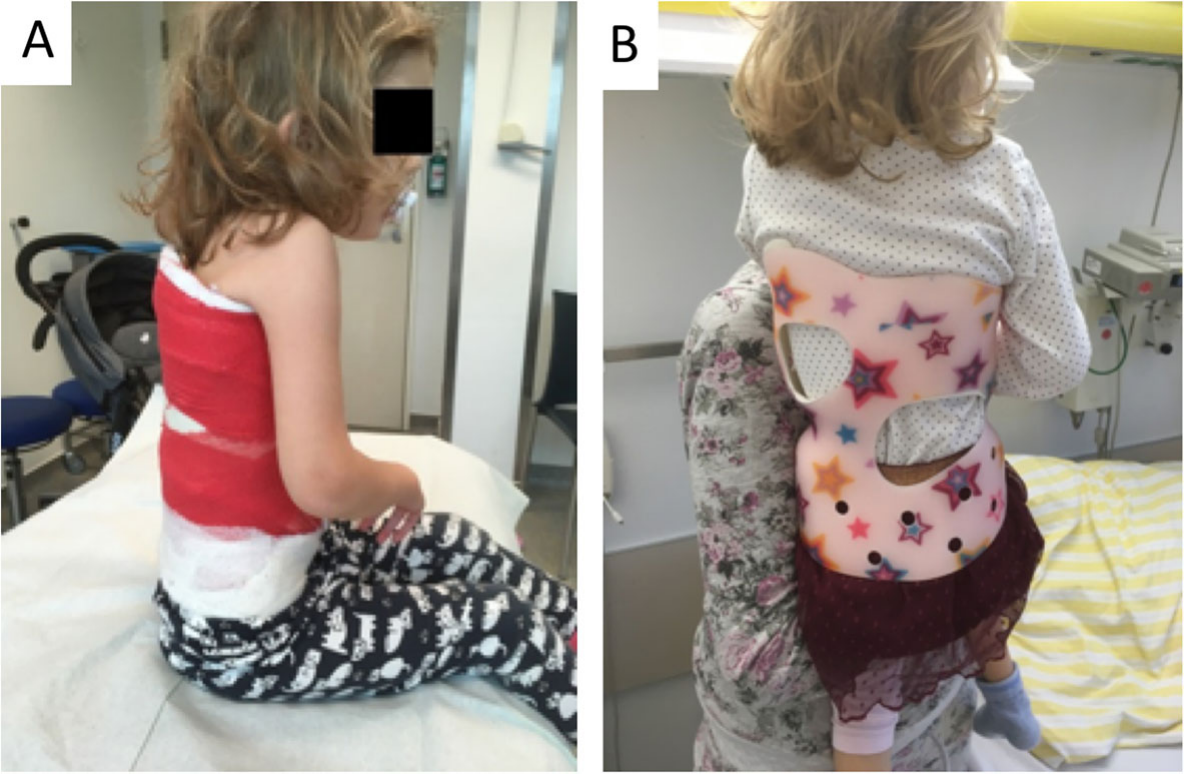
Example of a Risser cast and a modified Chêneau brace. Picture **a** shows a patient with SAS (age: 24 months) in a Risser cast. After the third Risser cast, a modified Chêneau brace was implemented. The brace was individually modulated according to the plaster cast (**b**). It was worn for 23 h per day. Clinical and radiological follow-up examinations were scheduled every 6 months

Staged correction was obtained by means of three consecutive casts changed at 4-week intervals. The casts were applied on a Risser table with the patient under general anesthesia using muscle relaxation. The correction maneuver consisted of longitudinal traction, derotation, and lateral pressure at the level of the apex with counterpressure at the level of the end vertebrae (Fig. 2). After completion of casting, a custom-made full-time Chêneau type brace was implemented (Fig. 3).

## Results

Sixteen patients fulfilled the inclusion criteria. There were 6 idiopathic and 10 syndromic curves. Altogether, 11 female and 5 male patients were included (male-female ratio: 1:2). The mean age at onset of treatment was 35 months (±15). The mean follow-up was 21 months (±15). The results were summarized in Table 1.

**Fig. 4:**
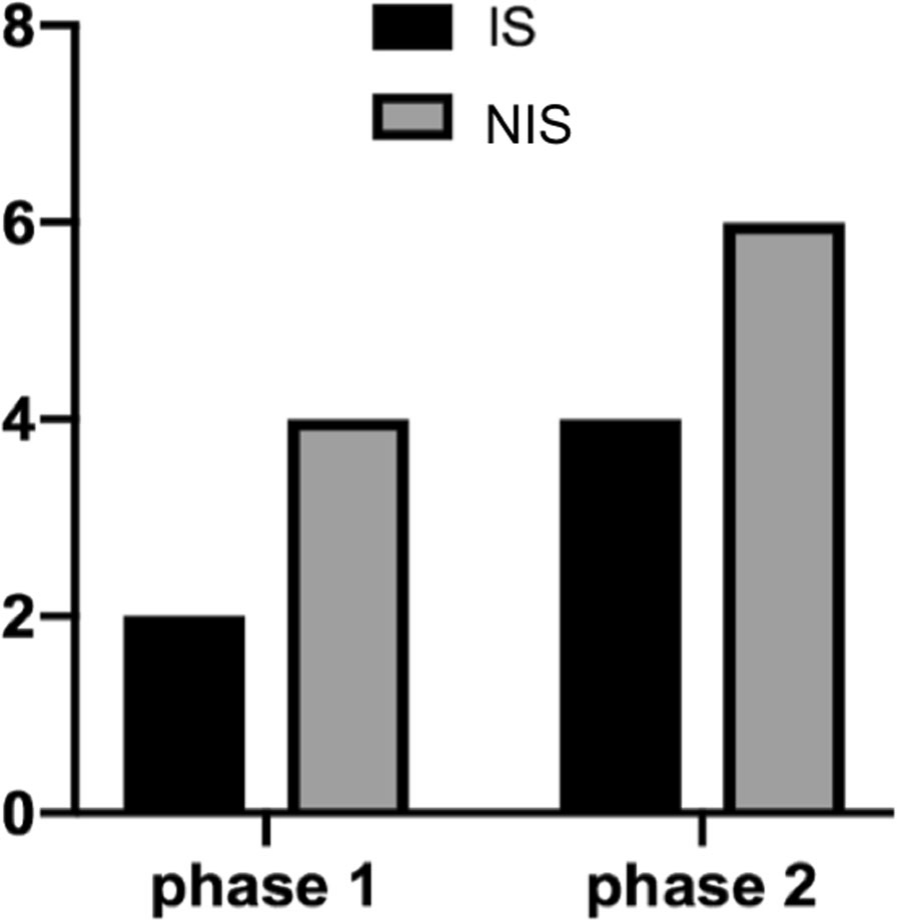
Distribution of the phase of rib-vertebra relationship. The response to treatment for scoliosis could be predicted by the rib-vertebra relationship according to Mehta. The distribution was fairly equal among the subgroups

In the IS cohort, two patients had a rib-phase 1 and four had a rib-phase 2. In contrast, in patients with NIS, four patients were in rib-phase 1 and six were in rib-phase 2 (Fig. 4).

In the IS cohort, curves had an average CA of 46°(±8) at the beginning of treatment and a mean RVAD of 20°(±12). After the first cast, a reduction to an average CA of 24°(±8) was achieved. After the third Risser cast, the curve was reduced to an average of 20°(±11), and a RVAD of 11°(±10) was observed. At the latest follow-up, an average CA of 16°(±7) and RVAD of 9°(±8) were measured with the patient in the Chêneau brace. The differences in CA from the third cast (*p* = 0.0017) and in the Chêneau brace (*p* = 0.018) were significant compared to treatment’s beginning. Furthermore, the RVAD was highly significantly reduced at the follow-up examinations compared to the treatment’s beginning of IS patients (*p* = 0.0046).

In patients with NIS, the mean CA at the onset of treatment was 55°(±15), and the mean RVAD was 24°(±14). Serial casting led to a consecutive reduction in CA. However, the reduction in RVAD was not statically significant. After the application of the third Risser cast, the mean CA was 28°(±12). This was a highly significant reduction in comparison to the beginning of treatment (*p* = 0.0019). The mean RVAD was reduced to 18°(±13). At the latest follow-up, an average CA of 31°(±11) was obsered. Thus, significantly less compared to the beginning of treatment (*p* = 0.0029). Average RVAD was 17°(±15) in the follow-up examinations of NIS patients.

**Fig. 5:**
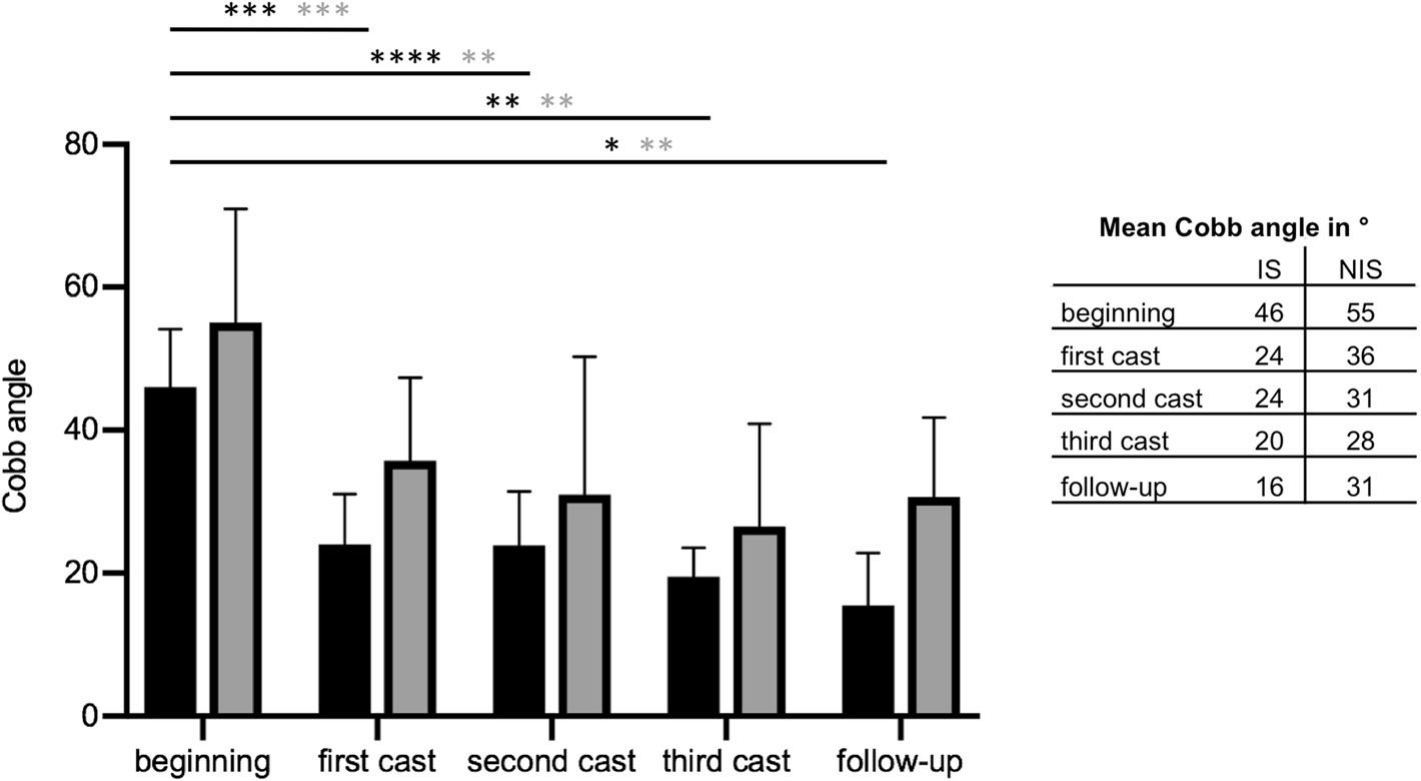
The graphs depict the CA at the beginning of treatment, during the serial casting period, and at the latest follow-up. The black columns depict the CA of patients with IS, and the grey columns represent the CA of patients with NIS. Likewise, statistical significance is indicated by black or grey *. With implementation of the first cast, a significant reduction in CA was achieved in both cohorts (IS *p* = 0.0009; NIS *p* < 0.0002). With the second cast, reduction in CA in relation to the CA at the beginning of treatment, was still significant (IS *p* < 0.0001; NIS *p* = 0.0036). Also, the third cast let to a reduction of significant reduction in CA, compared to the beginning of treatment (IS *p* = 0.0017; NIS *p* = 0.0019). In the follow-up controls the difference in CA was still significant to the beginning of treatment (IS *p* = 0.0182; NIS *p* = 0.0029). The error bars represent standard deviation

**Fig. 6:**
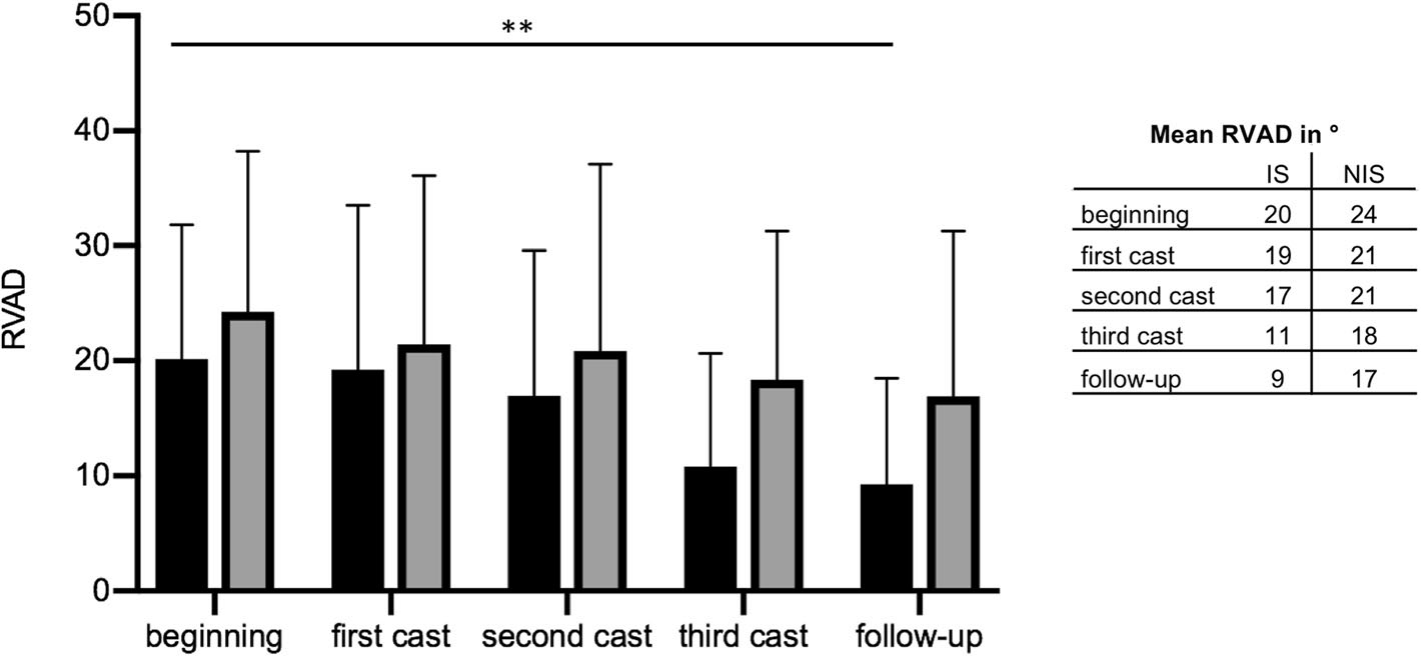
The graphs depict the RVAD at the beginning of treatment, during the serial casting period, and at the latest follow up. The black columns depict the RVAD of patients with IS, the grey columns depict the RVAD of patients with NIS. A significant reduction in RVAD was only observed in patients with IS, when compared from the beginning of treatment to the latest follow-up examination (*p* = 0.0046). However, no statistical difference in RVAD was seen between in patients with NIS. The error bars represent standard deviation

In summary, the mean correction of the CA was 65% in patients with IS compared to 44% in patients with NIS. The CA reduction was significant in both cohorts. Furthermore, reduction of RVAD was 55% in the IS group and 39% in the NIS group. A significant reduction in RVAD was only in IS patients achieved (Figs. 5 and 6).

No patients developed significant skin problems. Other complications like breathing or feeding problems were not observed. In neither case was the treatment abandoned due to compliance issues.

## Discussion

Our results support the treatment of EOS by serial Risser casting. The benefits for children with NIS of syndromic-associated origin are clear. A highly significant reduction in CA was achieved. The RVAD has proven as a reliable parameter to measure the complex apical three-dimensional spinal morphology on a plane radiograph [11]. Significant reduction in RVAD was only observed in IS patients. Whether the reduced RVAD through serial casting is as beneficial for the long-term course of scoliosis as it was observed for idiopathic “benign” EOS remains to be proven [12]. Due to prior serial casting, corrective spinal surgery can be delayed [13]. Poorer results from the treatment of NIS compared to IS can be explained by the more severe deformity at the onset of treatment (larger CA and RVAD).

An additional functional benefit is conferred by this treatment as well. Trunk elongation is accompanied by better trunk balance and reduction in falls [14]. The functional aspect needs to be emphasized for children with NIS. Because of the primary disease, these patients have fewer resources to compensate for pulmonary malfunction.

In our study, patients with syndromic curves responded to serial casting with an average correction of 24° (44%) in CA, which is significantly better than previously observed. We hypothesize that this can be explained by the more rapid changes in the casts with our treatment protocol (every 4 weeks). Sanders et al. performed serial casting for longer than 1 year [15]. Their approach involved changing the casts every 2 months in a 2-year-old and every 3 months in a 3-year-old child is not based on evidence and is not supported by any clinical observations. In our opinion, the prolonged cast treatment provides no advantages to the patient and may disproportionately increase complication rates in terms of skin breakage.

However, our practice as well as Sanders’ practice necessitate that young children receive multiple rounds of general anesthesia. The risks of multiple rounds of general anesthesia for the immature brain are not fully investigated, yet [16, 17]. From our point of view, correction and derotation at maximum can only be received with the help of a muscle relaxant agent. Unfortunately, Sanders did not give details regarding the anesthesia used in his study. But since he recommended intubation, the usage of a muscle relaxant agent is presumable.

But in general, the necessity of general anesthesia is under ongoing discussion. Similarities between serial castings and the Ponseti method for clubfoot treatment can be seen. Both methods base on redressing castings to correct a deformity. Comparable data with or without general anesthesia only exists for clubfoot treatment and revealed no difference in overall outcome [18]. The influence of general anesthesia and especially the influence of muscle relaxant agents on the outcome of correction in patients with serial casting should be further investigated.

Spontaneous resolution of IS has been observed in cases with the “benign” form [19]. In contrast, the natural history of non-idiopathic early onset scoliosis is usually progressive. As non-idiopathic EOS are curves referred to of a neuromuscular, congenital or syndromic origin. To take all these distinctively different pathologies together and put them into one clinical picture, is an obsolete idea. Therefore, we selected only patients with syndromic-associated EOS for comparison.

Because of the deteriorating nature of non-idiopathic EOS, most of the patients will develop curves over 80°, if left untreated. This results in significant thoracic deformity and extrinsic pulmonary restriction measured by diminished vital capacity [20]. Therefore, early treatment is mandatory. After the introduction of “growth-friendly” implants, surgical treatment with a non-fusion method has become the method of choice for patients with severe, progressive curves. However, higher complication rates after surgical treatment were observed in younger patients and in patients with NIS compared to those of idiopathic cases [21, 22]. Serial casting has been used as a safe and effective method for treatment of IS in order to correct a spinal curve or postpone surgical treatment [23]. Clinical experience with serial casting for the treatment of NIS is still lacking. Only recently, Baulesh et al. [24] and Gussous et al. [25] reported good results for serial casting in NIS patients. In contrast to our treatment protocol, the wear time and number of the casts were individually adapted for every patient [24] or the patients received five serial casts [25].

Baulesh observed a correction of only 5° in CA in patients with EOS of non-idiopathic origin, compared to 13° in IS patients treated by casting. Identical results have been published by Gussous with a correction of 5° in CA in patients with NIS, compared to 20° in IS patients.

Our results demonstrate an even greater reduction in scoliosis in syndromic-associated NIS patients compared to patients with IS by means of only three serial casts followed by consecutive implementation of a Chêneau brace.

The study has the following limitations: retrospective design and the short follow up. Many different protocols for serial casting in EOS patients exist and the very best has yet to be discovered. Therefore, prospective randomized studies are desirable.

## Conclusion

Serial casting is a good treatment option for EOS and should be considered in patients with syndromic curves. Our results show that in all cases, a significant curve correction was obtained and curve progression could be inhibited or decelerated in such a manner that the need for surgery could be delayed until the children were older. Syndromic etiology is no contraindication for serial casting in EOS.

## Data Availability

The raw data is available upon reasonable request from the corresponding author.

## Abbreviations

CA: Cobb angle
EOS: Early onset scoliosis
Fig: Figure
IS: Idiopathic early onset scoliosis
NIS: Non-idiopathic early onset scoliosis
RVAD: Rib-vertebra angle difference
